# Isolation, Characterization, and Stability of Discretely-Sized Nanolipoprotein Particles Assembled with Apolipophorin-III

**DOI:** 10.1371/journal.pone.0011643

**Published:** 2010-07-19

**Authors:** Nicholas O. Fischer, Craig D. Blanchette, Brent W. Segelke, Michele Corzett, Brett A. Chromy, Edward A. Kuhn, Graham Bench, Paul D. Hoeprich

**Affiliations:** Physical and Life Sciences Directorate, Lawrence Livermore National Laboratory, Livermore, California, United States of America; University of Milan-Bicocca, Italy

## Abstract

**Background:**

Nanolipoprotein particles (NLPs) are discoidal, nanometer-sized particles comprised of self-assembled phospholipid membranes and apolipoproteins. NLPs assembled with human apolipoproteins have been used for myriad biotechnology applications, including membrane protein solubilization, drug delivery, and diagnostic imaging. To expand the repertoire of lipoproteins for these applications, insect apolipophorin-III (apoLp-III) was evaluated for the ability to form discretely-sized, homogeneous, and stable NLPs.

**Methodology:**

Four NLP populations distinct with regards to particle diameters (ranging in size from 10 nm to >25 nm) and lipid-to-apoLp-III ratios were readily isolated to high purity by size exclusion chromatography. Remodeling of the purified NLP species over time at 4°C was monitored by native gel electrophoresis, size exclusion chromatography, and atomic force microscopy. Purified 20 nm NLPs displayed no remodeling and remained stable for over 1 year. Purified NLPs with 10 nm and 15 nm diameters ultimately remodeled into 20 nm NLPs over a period of months. Intra-particle chemical cross-linking of apoLp-III stabilized NLPs of all sizes.

**Conclusions:**

ApoLp-III-based NLPs can be readily prepared, purified, characterized, and stabilized, suggesting their utility for biotechnological applications.

## Introduction

Nanolipoprotein particles (NLPs) are nanometer-sized bilayer mimetics comprised of phospholipids and apolipoproteins [1], [2]. These discoidal particles are also referred to as recombinant high density lipoproteins (rHDLs) and nanodiscs. NLPs are ideally suited to solubilize, stabilize, and enable the study of membrane proteins [3]–[7], and provide an alternative to traditional membrane protein solubilization approaches including liposomes and detergent solubilization [8]. Although liposomes, polymeric particles, and nanoparticles have dominated the field of nanobiotechnology [9]–[14], NLPs represent a complementary biomimetic platform for many of the same applications. For example, NLPs have been used as drug delivery platforms [15]–[20], for diagnostic imaging [21], [22], and as vaccine platforms for the delivery of membrane protein [23] and recombinant subunit antigens [24].

NLP size, homogeneity, and stability are important parameters for biotechnology applications, such as membrane protein solubilization. In addition, the size of the NLP and the surface area of the lipid bilayer are key determinants of the potential for loading of cargo in or on the NLP bilayer. Homogeneity is important to provide readily characterizable starting material, and facilitates control over experimental parameters (e.g., stoichiometry). Understanding and characterizing NLP stability over time provides insight into shelf life of the NLPs, while also providing preliminary data on NLP integrity during *in vitro* and *in vivo* applications. As such, a clear understanding of both the methodology required to prepare discretely sized particles, as well as their inherent stability, is desirable.

The size of NLPs can be tuned in a number of ways. The choice of starting materials has a great effect, as different combinations of apolipoproteins and lipids have been demonstrated to generate a range of NLP sizes. For example, the human apolipoprotein apoA-1 has been extensively studied, and has been demonstrated to produce differently sized NLP species when prepared with either 1,2-dimyristoyl-*sn*-glycero-3-phosphocholine (DMPC) or 1-palmitoyl-2-oleoyl-*sn*-glycero-3-phosphocholine (POPC) lipids [25]. The identity of the apolipoprotein is also important, illustrated by the relatively large diameter NLPs produced with the 22 kDa fragment of human apoE4 (apoE422K), in contrast to the smaller apoA-I particles [26]. The ratio of lipid to apolipoprotein is also a major determinant in the ultimate size of the NLPs, with high ratios leading to the formation of larger NLPs [25], [27]. Incorporation of cholesterol at various molar ratios also affects particle size [28].

The goal of our research is to expand the repertoire of well-characterized lipoproteins currently available for biotechnological applications by demonstrating the facile preparation of discretely sized NLPs using insect apolipophorin III (apoLp-III), and to characterize their size, composition, and stability. ApoLp-III is primarily associated with lipid shuttling in hemolymph during insect flight, although additional roles in membrane remodeling and innate immunity have been identified [29]. ApoLp-III is a low molecular weight apolipoprotein (∼18 kDa) of high α-helical content (>70%), similar to vertebrate apolipoproteins. In particular, apoLp-III has high functional and structural similarity to the apoE422K [29], [30], which has been successfully adapted for biotechnological applications [3], [24], [31]. Importantly, and unlike other apolipoproteins, apoLp-III remains monomeric in the absence of lipid, even at high concentrations [29].

Herein we describe the successful assembly and purification of four NLP species of discrete diameter using apoLp-III. The two apoLp-IIIs chosen for this study were derived from insect species within the order Lepidoptera: *Manduca sexta* and *Bombyx mori.* These apolipoproteins are unglycosylated, small in size (166 and 164 residues, respectively), readily expressed and purified, share *ca.* 74% sequence identity [32], and have been well characterized [33], [34]. Four distinct NLP species readily form from apoLp-III incubated with DMPC and could be isolated to high purity by a single purification step using size exclusion chromatography. The NLP species were characterized by size exclusion chromatography (SEC), non-denaturing gradient gel electrophoresis (NDGGE), and atomic force microscopy (AFM). The stability of the individual NLP species assembled with *B. mori* apoLp-III was monitored for 5 months, which revealed significant remodeling of the smaller NLP species to a consensus structure with an average diameter of 20 nm. Chemical cross-linking was successful in stabilizing the individual NLP species for months. Although to date their use in biotechnological applications has been limited, apoLp-IIIs provide a means to expand the apolipoprotein toolkit for preparing NLPs featuring varied size ranges and characteristics.

## Materials and Methods

### Materials

DMPC was purchased from Avanti Polar Lipids (Alabaster, AL). All other reagents were ordered from Sigma-Aldrich (St. Louis, MO).

### Protein expression and purification

*B. mori* and *M. sexta* apolipophorin III constructs were generously provided by Dr. Robert Ryan. Proteins were expressed according to published procedures [33], [34]. *B. mori* apoLp-III concentrations were determined by UV spectroscopy at 280 nm in 3 M guanidine hydrochloride (ε_280_ = 6970 M^−1^ cm^−1^). *M. sexta* apoLp-III concentrations were determined with the Advanced Protein Assay Reagent (Cytoskeleton Inc., Denver, CO), using BSA as a standard.

### NLP assembly

NLPs were assembled according to previously reported procedures [2], [31], [35] with slight modifications. Briefly, lipids in chloroform were aliquoted into glass vials. Chloroform was then removed using a stream of N_2_ under agitation to form a thin lipid film, which was further dried *in vacuo* for at least 2 hours. Lipids were then solubilized in TBS buffer (10 mM Tris, 150 mM NaCl, pH 7.4) with 20 mM sodium cholate. After addition of apoLp-III, samples were incubated at 23.8°C for at least 4 hours. Final apoLp-III concentrations were *ca.* 150 µM during a typical assembly. Assemblies were then dialyzed against TBS to remove cholate and promote NLP self-assembly. Assemblies were subsequently analyzed and purified by SEC (Superdex 200, 10/300 GL column, GE Healthcare, Piscataway, NJ) in TBS buffer (0.5 mL/min flow rate, λ = 280 nm). Prior to SEC analysis, all samples were filtered using a 0.22 µm spin filter (Agilent) to remove any particulates. NLP fractions were analyzed by NDGGE and/or AFM. For NDGGE, 4–20% Tris-glycine polyacrylamide gels were used (Invitrogen, Carlsbad, CA). For all native gels, the NativeMark protein standard was used (Invitrogen). All gels were stained with SyproRuby (BioRad, Hercules, CA) and imaged using the Typhoon 9410 Variable Mode Imager (GE Healthcare). For remodeling studies, four large assemblies were prepared (175∶1, 135∶1, 75∶1, and 45∶1 lipid:apoLp-III molar ratios) to enrich the four different NLP populations (NLP-1, NLP-2, NLP-3 and NLP-4, respectively). To isolate the four different NLP populations, appropriate SEC fractions were pooled (**Figure S1A**). These fractions were stored at 4°C and, at indicated time points, a portion of each was analyzed by SEC, NDGGE, and/or AFM. For SEC chromatogram quantitation, a Nonlinear Least Squares Fitter (Origin 7, OriginLab Corp, Northampton, MA) was used to fit a multi-peak Gaussian function, corresponding to the appropriate NLP, lipid, or protein components. The integrated peak area was then used to assess relative abundance of each population.

### NLP composition analysis

Protein and lipid compositions were assessed for each purified NLP population. *B. mori* apoLp-III concentrations were determined by UV spectroscopy at 280 nm in 3 M guanidinium hydrochloride (ε_280_ = 6970 M^−1^ cm^−1^). DMPC concentrations were assessed using the colorimetric Phospholipids C kit purchased from Wako Chemicals USA, Inc. (Richmond, VA), following manufacturer's protocol using choline as a standard. For calculations of lipid bilayer area based on AFM-determined NLP diameters and DMPC molecular area, apolipophorin helices were assumed to contribute 2 nm to the overall diameter of the NLPs [36], [37]. To calculate lipid molecules per NLP, a published value of 0.6 nm^2^ per DMPC was used [38], [39] (DMPC/NLP = π(r_NLP_-1)^2^/0.6). After 162 days, the 20 nm NLPs in NLP-4 were purified by SEC for composition analysis.

### NLP cross-linking

For cross-linking experiments, NLP populations isolated by SEC were incubated with Bis-*N*-succinimidyl-(pentaethylene glycol) ester (henceforth abbreviated as PEO_5_). NLPs (*ca*. 25 ng apoLp-III per µL) in HEPES buffer (10 mM HEPES, 75 mM NaCl, pH 7.4) were incubated with 0, 0.5 and 5 mM PEO_5_ for four hours at room temperature. Reactions were quenched with 50 mM Tris-HCl (pH 7.4) for 30 minutes at room temperature. Samples were analyzed by NDGGE (4–20% Tris-glycine) and SDS PAGE (4–12% Bis-Tris with MES running buffer or 3–8% Tris-acetate with tricine running buffer). For denaturing gels, the Mark12 protein standard was used (Invitrogen).

### AFM analysis of NLPs

AFM measurements and analyses were conducted as previously described [31], [35], [36].

## Results

### NLP formation using *B. mori* and *M. sexta* apoLp-III

**Figure 1:**
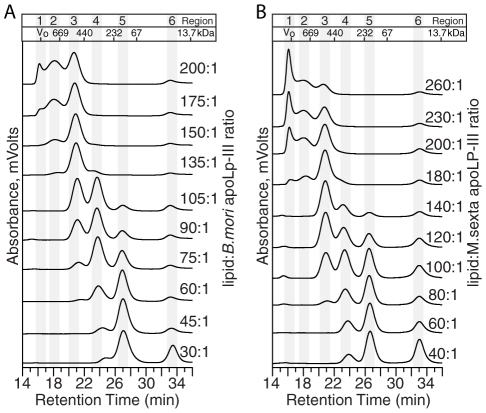
SEC chromatograms of NLP assemblies at various lipid-to-apoLp-III molar ratios. NLPs were assembled with DMPC and apoLp-III from A) *Bombyx mori* or B) *Manduca sexta*. SEC traces are parsed into 6 distinct regions: 1, lipid-rich; 2–5, NLPs; 6, protein-rich. Exclusion limit of the column (V_o_) and molecular weights of protein standards are indicated.

Discoidal nanolipoprotein particles have been prepared using purified apoLp-III from myriad species, including *Locusta migratoria*, *M. sexta*, and *B. mori* [33], [40], [41]. To determine the potential use of apolipophorin-based NLPs for biotechnology applications, NLPs assembled using apoLp-III from *B. mori* and *M. sexta* were investigated. Various lipid:apoLp-III ratios were surveyed for successful NLP formation. ApoLp-III was incubated with DMPC in the presence of cholate for *ca.* 4 hours at 23.8°C. After dialysis of the assembly reactions to remove excess cholate, samples were analyzed by SEC. The resultant chromatograms were effectively parsed into 6 distinct regions (**Figure 1**), corresponding to lipid-rich complexes (Region 1, ∼16 min), four distinct NLP peaks (Regions 2 to 5, ∼18 to 28 min), and protein-rich species (Region 6, ∼33 min). Identical peaks were observed in assemblies containing *B. mori* and *M. sexta* apoLp-III. Assemblies at high lipid:protein ratios were predominated by large, lipid-rich complexes eluting in the column void volume (∼16 min) and two NLP species (∼18 and ∼21 min). Smaller NLP species were observed (∼23.5 and 27 min) at lower lipid ratios. As the lipid:protein ratios were decreased, the amount of unincorporated protein increased (∼33 min), suggesting that not enough lipid was present to utilize all the protein for NLP formation. These results resemble the well-established phenomenon that NLP size can be controlled by lipid:apolipoprotein stoichiometry [25], [27]. While apoLp-III derived from both *M. sexta* and *B. mori* successfully formed NLPs, the latter was chosen for further in-depth analysis. The primary reason for this was the difficulty in the detection and quantitation of *M. sexta*-derived NLPs; the protein has only a single tyrosine residue (and no tryptophan) and hence low intrinsic absorbance.

### Identification of discrete NLP species

To verify that the particles isolated from the SEC regions 2 to 5 were indeed NLPs, two independent techniques were used to characterize the *B. mori* assemblies; NDGGE and AFM. NDGGE is a traditional analytical technique used to differentiate between NLPs of varying sizes according to electrophoretic mobility and apparent molecular weight. AFM provides single-particle analysis of NLP diameter and height.

**Figure 2:**
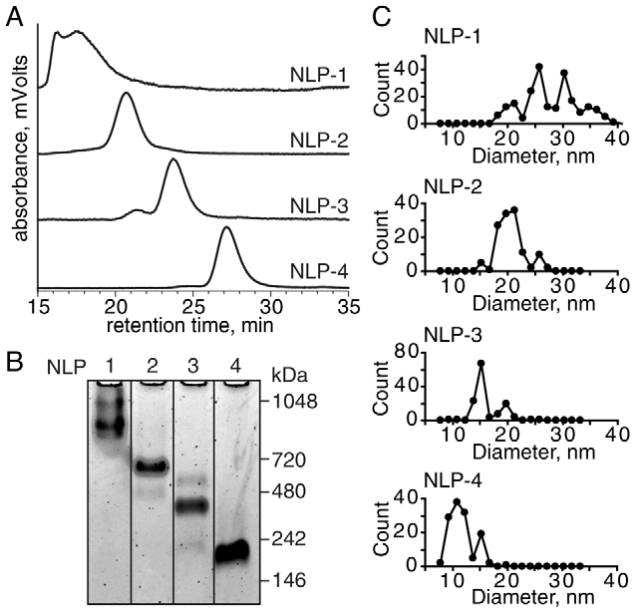
Characterization of purified *B. mori* apoLp-III NLP species. A) SEC analysis of purified NLPs. B) NDGGE analysis of purified NLPs. C) AFM size distributions for each population are shown (n>120, 1.5 nm binning intervals). Representative AFM images are shown in Figure S2.

First, four assembly conditions (i.e., molar lipid:protein ratios) were identified from **Figure 1** that enriched a particular NLP species. The lipid:apoLp-III molar ratios used to form NLP species of different sizes were 175∶1, 135∶1, 75∶1 and 45∶1 (**Figure S1**). The 175∶1 ratio produced large NLPs with a retention time of ∼18 minutes, henceforth referred to as NLP-1. NLP-2 denotes the species with a retention time of ∼21 minutes, and was the dominant species produced using a 135∶1 molar ratio. Molar ratios of 75∶1 and 45∶1 produced enriched NLP species at ∼23 and ∼27 minutes, respectively, termed NLP-3 and NLP-4. The fractions corresponding to the desired NLP species were pooled and reanalyzed by SEC (**Figure 2A**) to demonstrate purity. All subsequent characterization was conducted on these purified NLPs (NLP-1, NLP-2, NLP-3, NLP-4). NDGGE demonstrated that each SEC peak corresponded to a discrete NLP species, except NLP-1 (**Figure 2B**). NLP-1 was characterized by two dominant and multiple faint bands, suggesting multiple NLP species ranging in apparent molecular weight (MW) from ∼950 to 1100 kDa (**Table 1**). This was not unexpected, as the SEC column used in these studies had limited resolution for particles with apparent MW in this range. The remaining three NLP species were dominated by species at *ca.* 560, 360, and 220 kDa (NLP-2, NLP-3, and NLP-4, respectively). To further verify the presence of NLPs, AFM was used to confirm the discoidal morphology of the purified species (**Figure S2**). Single particle analysis by AFM elucidated that NLP-1 was comprised of numerous different sized NLP species (**Figure 2C**), corroborating the presence of multiple bands by NDGGE. The two dominant species were 25.4±0.9 nm and 30.5±1.1 nm in diameter; two minor species were 20.0±1.6 and 35.1±1.3 nm. The other three NLP species were each characterized by a single dominant species with average diameters of 20 nm, 15 nm, and 10 nm (NLP-2, NLP-3, and NLP-4, respectively) (**Figure 2C** and **Table 1**). The heights of the three largest purified NLP species (NLP-1, NLP-2, and NLP-3) were *ca.* 6.2 nm, whereas the smallest NLP-4 species was 5.2 nm.

**Table 1:** Summary of NLP characteristics.

	NLP-1	NLP-2	NLP-3	NLP-4
AFM diameter (nm) a	≥25 g	20.0±1.6	14.9±0.8	10.7±1.1
AFM height (nm)	6.1±0.3	6.3±0.4	6.1±0.5	5.2±0.5
Apparent MW (kDa) by SEC	1015	556	263	116
Apparent MW (kDa) by NDGGE	930, 1040	561	365	217
Input DMPC/apoLpIII b	175	135	75	45
# DMPC/apoLpIII c	127.2±20.9 h	111.6±8.7	63.5±13.2	53.3±7.3
# apoLpIII/NLP d	≥5 i	4	3	2
DMPC/NLP e	636±105	447±35	190±40	107±15
DMPC/NLP f	717±55	424±75	217±27	99±25

*^a^*Calculated from full-width half-maximum (FWHM).

*^b^*Molar lipid-to-protein ratio.

*^c^*DMPC quantified using enzymatic assay. ApoLp-III quantified by UV-Vis spectroscopy.

*^d^*Number of apoLp-III molecules determined by chemical cross-linking and SDS gel visualization.

*^e^*Calculated using experimentally determined DMPC/apoLp-III and apoLp-III/NLP.

*^f^*Calculated based on measured AFM diameters and DMPC molecular area, assuming 0.6 nm^2^/DMPC and 1 nm apoLp-III helix thickness. DMPC/NLP = π(r_NLP_-1)^2^/0.6.

*^g^*NLP-1 comprised of three main species: 25.4±0.9 nm, 30.5±1.1 nm, and 35.1±1.3 nm.

*^h^*Number of DMPC lipid molecules per NLP was determined for aggregate of all NLP-1 sizes.

*^i^*25 nm, 30 nm, and 35 nm NLP-1 species hypothesized to contain 5, 6 and 7 apoLp-III molecules, respectively.

### Compositional analysis of NLP species

**Figure 3:**
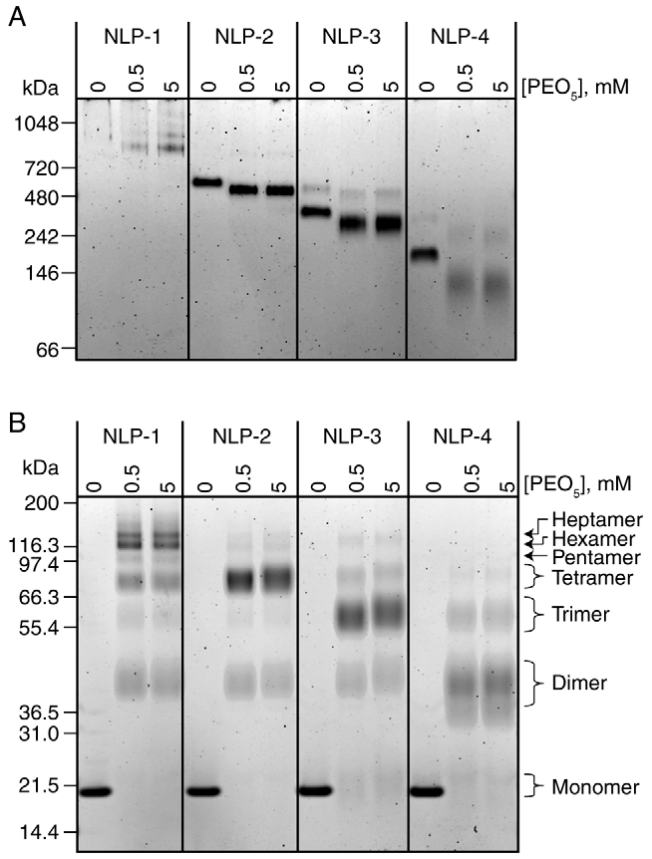
Gel analysis of cross-linking experiments. Purified B. mori apoLp-III NLP species were cross-linked with indicated concentrations of PEO_5_ and analyzed by gel electrophoresis. A) NDGGE (4–20% Tris-glycine) demonstrates that NLPs maintain integrity upon cross-linking. B) Denaturing SDS PAGE (4–12% Bis-tris gel) bands correlate to number of apolipoprotein molecules. To verify mobility of high molecular weight bands, a 3–8% Tris-acetate SDS gel was run (see Figure S3).

The four purified NLP species were analyzed to determine the lipid-to-apolipophorin ratios as well as the number of apolipophorin molecules per NLP. The composition of each NLP species was elucidated by independently determining the lipid and protein concentrations of purified NLPs, providing molar ratios of the NLP constituents (see experimental section for details). As summarized in **Table 1**, the lipid:protein ratio increased as the size of the NLPs increased. The number of apoLp-III molecules in each of the four NLP species was elucidated by chemically cross-linking available free amines on the apoLp-III surface using the symmetric cross-linker PEO_5_. To avoid inter-NLP cross-linking, dilute NLP concentrations were used for these studies. By NDGGE (**Figure 3A**), cross-linked NLPs remained intact and exhibited slightly faster mobility, presumably due to modification of the NLP surface charge due to cross-linking through solvent-exposed lysine residues. Importantly, no higher molecular weight bands were observed in the cross-linked species, indicating that inter-particle cross-linking did not take place or had undetectable occurrence. When analyzed on denaturing gels, different dominant bands corresponding to cross-linked apoLp-III were observed for each NLP species (**Figure 3B**). Higher molecular weight bands were observed as the size of the NLP increased. For example, cross-linked NLP-1 displayed intense bands corresponding to five, six and seven cross-linked apoLp-III molecules. NLP-2, 3, and 4 displayed bands that correlated to primarily four, three, and two cross-linked apoLp-III molecules, respectively (**Table 1**). No difference in cross-linking efficiency was observed between 0.5 and 5 mM PEO_5_, suggesting that the cross-linking reaction was complete. To elucidate the higher molecular weight species corresponding to multiple cross-linked apoLp-III molecules, samples were separated on a 3–8% Tris-acetate gel (**Figure S3**) and the results correlated well with those shown in **Figure 3B**. Based on the number of apolipophorin molecules in each NLP and the lipid:protein ratio determined by lipid and protein quantification, the total number of lipid molecules contained within the NLP bilayers were calculated to be 636±105, 447±35, 190±40, and 107±15 for NLP-1, NLP-2, NLP-3, and NLP-4, respectively (**Table 1**). Independent estimates of the number of lipid molecules per NLP based on particle diameter and molecular area of the DMPC lipid headgroups were within the margin of error determined by compositional analysis. Assuming an average DMPC molecular area of 0.6 nm^2^ [38], [39], and an apoLp-III helical thickness of 1 nm [37], the average number of DMPC molecules per NLP extrapolated from the NLP diameter as measured by AFM were 717±55, 424±75, 217±27, and 99±25 nm for NLP-1, NLP-2, NLP-3, and NLP-4, respectively (**Table 1**).

### Dynamic remodeling of NLP species

NLPs were monitored at various time points during storage and stability was assessed by both bulk and single molecule analyses. NLPs of each size were purified by SEC, stored at 4°C, and periodically assessed over 5 months. SEC and NDGGE were used to measure changes in the average characteristics of the bulk sample, whereas AFM was used for single particle measurements.

**Figure 4:**
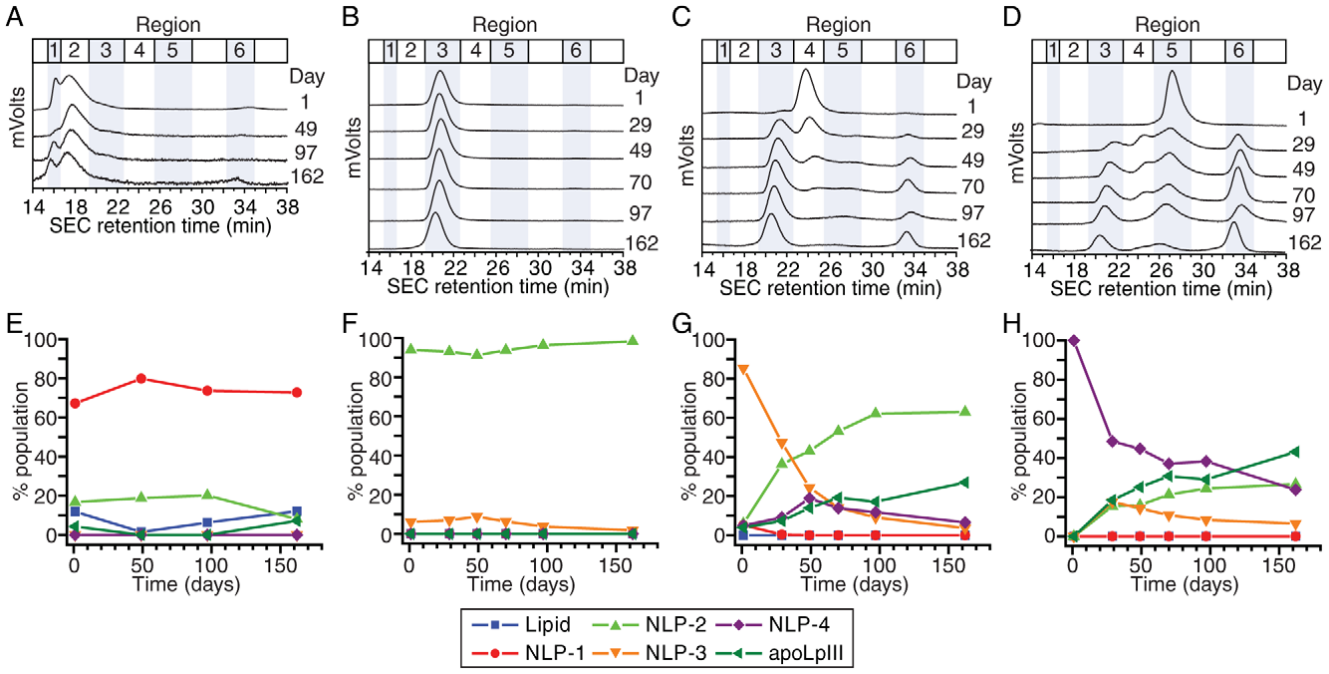
SEC analysis of NLP remodeling. Purified *B. mori* apoLp-III NLPs were stored at 4°C and monitored over 5 months by SEC. A–D) Raw SEC chromatograms were quantified by multi-peak integration of SEC chromatograms. E–H) Relative abundance of individual components corresponding to NLP peaks (NLP-1, NLP-2, NLP-3, NLP-4) or non-NLP peaks (lipid- or protein-rich species) is represented as percent of total chromatogram signal integration. A,E) NLP-1, B,F) NLP-2, C,G) NLP-3, and D,H) NLP-4. Nomenclature of remodeled NLPs was assigned according to retention time (NLP-1, Region 2, 18 min; NLP-2, Region 3, 21 min; NLP-3, Region 4, 23.5 min; NLP-4, Region 5, 27 min). Lipid- and protein-rich peaks had retention times of 16 (Region 1) and 33 min (Region 6), respectively.

Each purified NLP species was assessed by SEC at periodic intervals to determine the purity of the samples. If remodeling occurred, a decline in the overall percentage of the originally isolated and purified NLP would be observed, along with a concomitant increase in the amount of NLPs of other sizes. The SEC chromatograms were analyzed to assess relative abundance of each NLP species, as well as the lipid- and protein-rich components (**Figure 4**). Nonlinear least squares fitter (Origin 7, OriginLab Corp.) was used to fit the multiple peaks of the chromatograms to Gaussian functions, facilitating the integration of the six chromatogram regions. NLP-1 demonstrated little change in overall abundance of the main peak centered at 18 min (**Figure 4A,E**). Similarly, NLP-2 remained unchanged over the 5 month period (**Figure 4B,F**). In contrast, a dramatic remodeling of the originally isolated NLP species was seen in the smaller NLP-3 and NLP-4 species. In the case of NLP-3, a rapid disassembly of the NLP-3 particles occurred, which was mirrored by concomitant increases in both NLP-2 particles and free protein (**Figure 4C,G**). By 50 days, NLP-2 was the dominant NLP species and remained so for the duration of the study. Similarly, the NLP-4 population underwent significant remodeling (**Figure 4D,H**). Within 30 days, over 50% of the NLP-4 particles had disassembled, and an increase in both NLP-2 and NLP-3 species was observed. Again, a rise in free Lp-III was observed. By the end of the study, the sample was comprised of similar percentages of the NLP-2 and NLP-3 particles. Identical results were observed by NDGGE (see **Figure S4**).

To determine if NLPs of a particular size formed by remodeling were identical to those initially purified from an assembly, NLP-2 species remodeled from NLP-4 (after 162 days) were isolated by SEC. The purified NLP-2 population was then analyzed for DMPC and apoLp-III content. The composition of NLP-2 formed through remodeling of the NLP-4 species was identical to the stable NLP-2 particles originally purified from the NLP assembly (110.9±18.7 DMPC/apoLp-III vs. 111.6±8.7 nm DMPC/apoLp-III, respectively).

**Figure 5:**
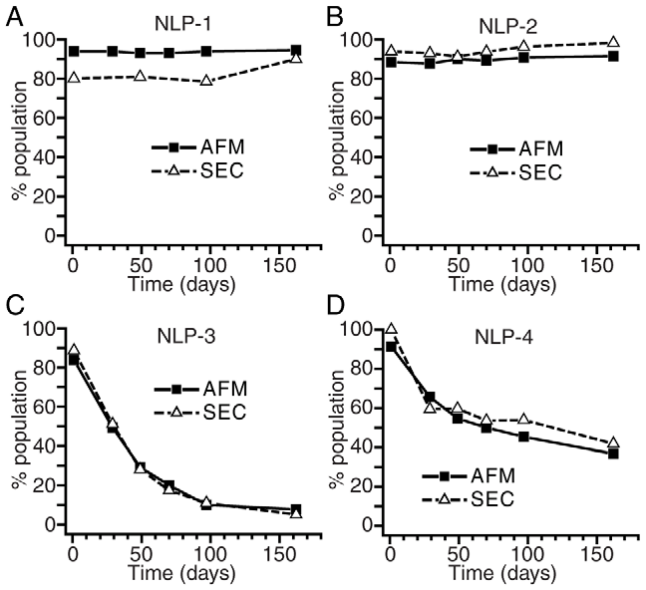
Comparative assessment of *B. mori* apoLp-III NLP remodeling by AFM and SEC. Identical trends in the fate of the four purified NLP species stored over 5 months at 4°C are elucidated by both single molecule (AFM) and bulk (SEC) measurements. A) NLP-1, B) NLP-2, C) NLP-3, and D) NLP-4. For representation of AFM data, nomenclature of remodeled NLPs was assigned according to diameter (NLP-1, ≥25 nm; NLP-2, 20 nm; NLP-3, 15 nm; NLP-4, 10 nm).

Single particle measurements using AFM gave results that were consistent with bulk measurements. AFM was used to measure NLP diameters, which allowed us to assess the distribution of NLP species. As free protein and lipid were not readily measurable by AFM, a direct comparison between AFM and SEC was afforded by looking at the distribution of NLP species as a fraction of total NLPs. For representation of AFM data, NLPs were assigned according to diameter (NLP-1, ≥25 nm; NLP-2, 20 nm; NLP-3, 15 nm; NLP-4, 10 nm). The relative abundance of each NLP species at each time point was nearly identical between AFM and SEC (**Figure 5** and **Figure S5**).

The single particle measurements by AFM elucidated population distributions in NLP-1 that were undetected by SEC (due to resolution limitations of the SEC column used in this study) (**Figure S6**). By SEC, all NLP-1 chromatograms featured a dominant peak at 18.2 minutes, suggesting no change in NLP-1 size. However, the ability to interrogate individual NLPs by AFM provided greater resolution to measure larger particles. On Day 0, the 25 nm NLPs were the dominant species, followed closely by 30 nm and 35 nm NLPs. By Day 25, 30 nm NLPs were the majority species, although significant amounts of the other NLP species were still present. After 150 days, however, the only true NLP species (i.e., particles with diameters less than 37.5 nm) present in significant quantities were the 30 nm particles. Although NLPs larger than 37.5 nm could be present, AFM is not able to distinguish these larger NLPs from these from adhered liposomes [42], [43].

### Stability of cross-linked NLP species

**Figure 6:**
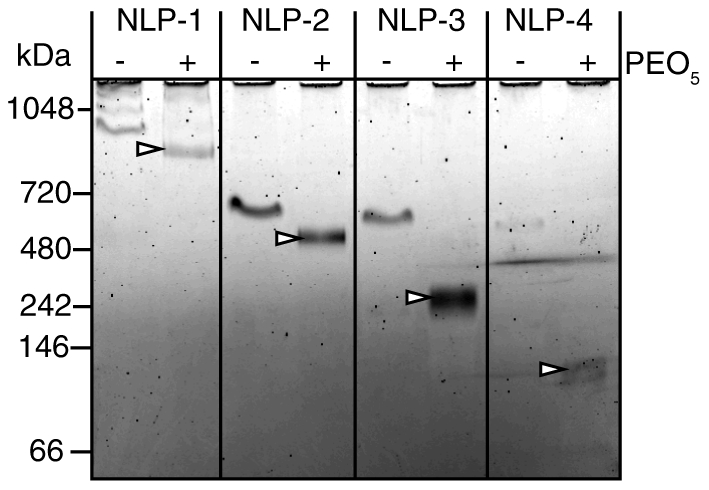
Stability analysis of cross-linked *B. mori* apoLp-III NLPs stored for two months at 4°C. Cross-linked and non-cross-linked samples (0.5 mM PEO_5_) were analyzed by NDGGE. Stable cross-linked species are indicated by arrowheads.

The dynamic remodeling, or instability, of NLPs can present difficulties for applications requiring stable NLPs of discrete sizes. To address this, we investigated the possibility of increasing the stability of NLPs by chemically cross-linking free reactive amines available on the apoLp-III protein surface. While cross-linking of lipoprotein constituents has been done for decades to identify the number of lipoprotein molecules per particle, to our knowledge the subsequent stability of these particles has never been studied. Cross-linked and non-cross-linked NLPs were stored at 4°C and analyzed after two months by NDGGE (**Figure 6**). Again, the cross-linked NLPs exhibited slightly greater mobility than the non-cross-linked NLPs, presumably due to modification of the NLP surface charge due to cross-linking through solvent-exposed lysine residues. As anticipated, the non-cross-linked NLP-1 and NLP-2 species were still present, whereas NLP-3 and NLP-4 were no longer present. Conversely, the cross-linked NLP species exhibited discrete bands that were identical to the starting samples (compare **Figure 6** to **Figure 3**), indicating that particle size persisted. These data suggest that the NLPs can be stabilized through chemical cross-linking. Significant stability of cross-linked NLP was also observed after one year of storage, although some degradation was evident (data not shown). These results demonstrate that chemical cross-linking provided a means to stabilize the 10 and 15 nm NLP species. The non-cross-linked 20 nm NLP-2, on the other hand, was completely stable after one year of storage, indicating that further stabilization of that species by cross-linking was not necessary.

## Discussion

The versatile nature of the NLP assembly process readily accommodates a diverse range in both the lipid [26], [31], [44], [45] and protein [26], [29], [46] constituents. Importantly, the choice of starting material and assembly conditions provides the ability to control the purity, size, and stability of the NLPs. The majority of NLPs used for biotechnology applications have utilized human apolipoproteins, in particular apoA-1 [15], [22], [45] and apoE4 [3], [24], [31]. As the identity of apolipoprotein has an impact on NLP size and stability, characterizing NLPs formed with additional apolipoproteins expands the repertoire of NLP building blocks. To this end, insect apolipophorins represent excellent candidates as they can be readily expressed and purified, and are well characterized [33], [40], [41].

NLPs have been identified as platforms for many biotechnological applications, including therapeutics and diagnostics [15]–[22], [24], [31]. Furthermore, the use of NLPs for membrane protein solubilization and stabilization has greatly expanded in recent years [3]–[7], [47]. This technology has now been optimized for broad applicability, as epitomized by Invitrogen's MembraneMax™ commercially available protein expression kit based on *in vitro* protein translation. Specifically for membrane protein solubilization, NLP size homogeneity is an important factor for a variety of applications. In particular, structural characterization of membrane proteins is greatly facilitated by homogeneous NLP populations as well as control over the number of membrane proteins incorporated into the NLP bilayer. A means of systematically controlling both size and stability of the NLPs, as described herein, would be of great benefit to this technology.

### Characterization of discretely sized NLP species

NLPs were successfully prepared using apolipophorin III from two insect species, *B. mori* and *M. sexta*. Varying the lipid to apoLp-III ratios resulted in the formation of four distinct NLP sizes elucidated by SEC. These distinct NLP populations could be readily isolated to a high degree of purity by a single SEC purification step. In contrast, purification of apoA-I-derived NLPs is not easily achieved by SEC, and requires a more laborious approach using ultracentrifugation [25], [27]. Using NDGGE and AFM, the presence of four distinct NLP species were identified (**Table 1**). NLP-1, while appearing as a single species by SEC, was more adequately resolved by NDGGE and AFM. By NDGGE, NLP-1 was a high molecular weight smear, represented by three to four faint bands. AFM was able to resolve the presence of three uniquely sized NLP species with diameters of *ca.* 25, 30 and 35 nm. Although objects of large diameter were observed, AFM was not able to distinguish these from adhered liposomes, which can be as small as 40 nm in diameter [42], [43]. Interestingly, the height of the small NLP-4 species is almost 1 nm shorter than all other *B. mori* NLPs (**Table 1**). This may suggest that lipid packing or overall structure of the small NLPs may be quite different than the larger NLPs. A number of studies have reported that NLPs of smaller diameters may not assume a discoidal morphology characterized by a planar bilayer [48]. Rather, these particles assume a saddle-shaped structure exhibiting a minimal surface [49], [50]. Alternatively, the interaction forces between the mica surface and particle may be different when the NLPs are this small, potentially affecting the thickness of the intervening water layer. Addressing this discrepancy merits further investigation.

### NLP remodeling

A number of groups have investigated the spontaneous remodeling of apoA-I-based rHDLs over time [25], [51]–[53] as well as resulting from actions by enzymes such as phospholipid transfer protein [54], lecithin:cholesterol acyltransferase [55], and cholesteryl ester transfer protein [56]. Although anticipated based on the observations made with apoA-I, no evidence of the remodeling of apoLp-III based particles has been reported.

As the stability of discretely sized NLP species is important for most potential applications, we examined the stability of apoLp-III NLPs of different sizes over time. In contrast to SEC, AFM demonstrated that remodeling of NLP-1 indeed occurred, resulting in a predominance of 30 nm particles by the end of the test period. An increase in particles greater than 37.5 nm in diameter may suggest that a mechanism for producing the most stable 30 nm species involves the formation and dissociation of much larger structures. The inability of the AFM to adequately resolve these very large structures as NLPs, however, limits the ability to draw firm conclusions from these data. NLP-2 displays no remodeling over the entire test period at 4°C, indicating that the 20 nm NLPs comprised of 4 Lp-III molecules were very stable. Indeed, NLP-2 samples stored for 13 months were completely homogeneous and exhibited no remodeling by NDGGE (data not shown). The stability of NLP-2 species was also demonstrated by the time-dependent remodeling of the smaller NLP species, NLP-3 and NLP-4. NLP-3 exhibited minimal stability over time, remodeling to form predominantly the 20 nm NLP-2, accompanied by the appearance of free apoLp-III. Remodeling of the NLP-3 may involve NLP-4 as an intermediate, as evidenced by the appearance of a significant fraction of NLP-4 by 50 days. NLP-4 also demonstrated rapid remodeling, resulting in predominantly NLP-2 and free apoLp-III. NLP-3 may play a role as an intermediate during the remodeling process, peaking at ∼20% by 29 days. These data suggest that NLP-2 species are the most stable, and that smaller NLPs will ultimately rearrange to form NLP-2. This is corroborated by the compositional analysis of the 20 nm particle (NLP-2) formed by remodeling, indicating nearly identical molar DMPC to apoLp-III ratios as NLP-2 particles directly purified from an assembly reaction. When experiments were conducted at 25°C, similar remodeling profiles were observed (data not shown). NLP-3 and NLP-4 remodeled to form predominantly the 20 nm NLP-2, although the remodeling time scales were accelerated 5-fold. The stability of NLP-2 decreased as well, and exhibited a half-life of approximately 15 days (data not shown).

**Figure 7:**
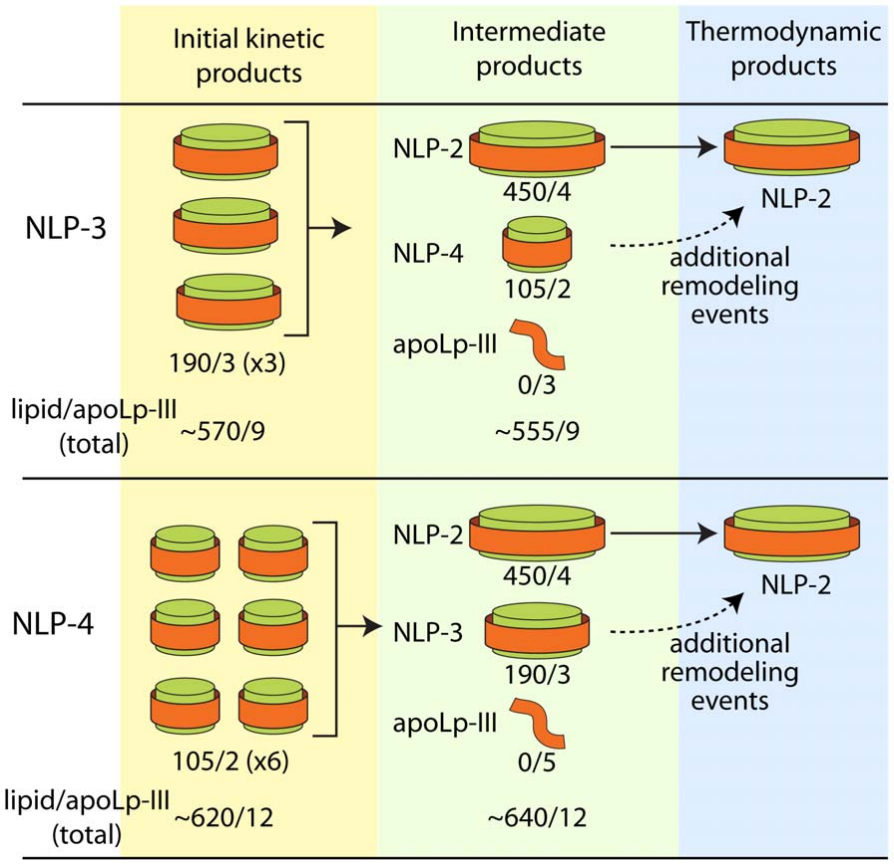
Mechanism of NLP-3 and NLP-4 remodeling. Initial kinetic products are formed by self-assembly at defined apoLp-III and DMPC ratios. Over time, both NLP-3 and NLP-4 form intermediate species, ultimately remodeling into predominantly the 20 nm NLP-2 species. The numbers of lipid and apoLp-III molecules per NLP species are indicated.

The remodeling data suggest that total lipid in the system is the limiting factor for the remodeling of the NLP-3 and NLP-4 species. Free apoLp-III accumulated as more of the smaller NLPs remodeled into NLP-2, while no free lipid was observed. The remodeling of the smaller NLP species follows a complex mechanism, involving the disassembly and reassembly of the three different NLP species (i.e., NLP-2, -3, and -4), ultimately resulting in a preponderance of the 20 nm NLP-2. As demonstrated in **Figure 4**, the path from NLP-3 to NLP-2 involved an NLP-4 intermediary. Similarly, NLP-3 was transiently formed as NLP-4 remodeled to NLP-2. As outlined in **Figure 7**, the intermediate NLPs are a proposed consequence of the disassembly of the initial kinetic products to ultimately form the thermodynamically stable NLP-2 species. In all cases, lipid appears to be the limiting reagent. In the proposed remodeling scheme (and supported by experimental evidence), NLP disassembly results in free apoLp-III. This is anticipated, as the smaller NLP species have much lower lipid-to-apoLp-III ratios (53∶1 and 63∶1, for NLP-4 and NLP-3, respectively) than NLP-2 (ca. 110∶1) (Table 1). To illustrate this, the remodeling schemes of NLP-3 and NLP-4 are depicted in **Figure 7**. The 15 nm NLP-3 species represents a kinetic minimum when DMPC and apoLp-III are initially assembled at a 75∶1 ratio. Over time, however, a more thermodynamically stable structure is produced (NLP-2), accompanied by a small fraction of transient NLP-4 species. For example, 3 NLP-3 particles are needed to satisfy the total lipid amount needed for one NLP-2 particle (450 DMPC molecules). The remaining 5 free apoLp-III molecules and ca. 120 lipid molecules can then reassemble into a single NLP-4 species (comprised of 2 apoLp-III and ca. 105 DMPC molecules). These intermediate NLP-4 particles will ultimately remodel to form the NLP-2 species. A similar mechanism can be described for NLP-4 (**Figure 7**). The disparity between kinetic and thermodynamic NLP products may be attributed to elasticity of apolipoprotein conformations. For example, human apoAI has been shown to assume myriad conformations, contributing to an overall broad conformational space [57], [58] that is dependent on temperature and constituent ratios. These results are consistent with the observed stability of cross-linked NLPs, whereby the conformational space of the apoLp-III is greatly limited due to the covalent inter-apoLp-III cross-linking within each NLP.

### Bulk vs. single molecule measurements

The combination of bulk and single-molecule analyses is a powerful tool for the study of NLPs. The strong correlation between the AFM and SEC monitoring of NLP remodeling is important (**Figure 5** and **Figure S5**), as it validates the utility of both techniques in the study of NLPs. Furthermore, the tandem use of these techniques provides additional information beyond use of a single technique. The analysis of bulk characteristics using SEC provides insight into the average behavior. Importantly, SEC provided information of the presence of both lipid-rich and protein-rich species. This compensated for a limitation of AFM, namely the inability to easily interrogate single proteins on the mica surface. Conversely, AFM provided data on larger NLPs that were beyond the resolving capability of the SEC column used in this study. Had SEC alone been used to monitor the NLP-1 species, the remodeling of larger NLPs to 30 nm particles would not have been elucidated.

### Conclusion

In conclusion, we have demonstrated the ability to tailor the size of lipophorin-based NLPs. Four discrete NLP species with diameters of 10 nm, 15 nm, 20 nm, and ≥25 nm can be readily assembled, purified to homogeneity by SEC, and characterized. Bulk and single-particle analyses demonstrated that the 15 nm and 10 nm NLP species undergo spontaneous remodeling over time. However, the 20 nm NLP were extremely stable, and demonstrated no remodeling even when stored for over 1 year at 4°C. While smaller NLPs had only limited stability, chemical cross-linking can extend NLP stability to many months. These finding provide a knowledge base for the rational design of lipophorin NLPs for biotechnology applications.

## Supporting Information

Figure S1 Isolation of B. mori apoLp-III NLP species. A) To isolate the various NLP peaks observed by SEC, four lipid:protein ratios were chosen to provide significant enrichment of the individual peaks (NLP-1, 175:1; NLP-2, 135:1; NLP-3, 75:1; NLP-4, 45:1). SEC fractions (shaded regions) for each NLP peak were pooled for further analysis. B) Pooled fractions from (A) were reanalyzed by SEC, demonstrating purity of the NLP species.
(0.20 MB TIF)

Figure S2 Representative AFM micrographs of the four B. mori apoLp-III NLP species purified by SEC. Scale bars correspond to 50 nm. Full-width half-maximum analysis of NLP diameter is represented by the green area in the pseudo-colored image, which accounts for the tip convolution effect. The slow scan direction (vertical) was used for particle diameter analysis.
(1.11 MB TIF)

Figure S3 Gel analysis of crosslinking experiments. SDS-PAGE (3–8% Tris-acetate, MES running buffer) was used to resolve high molecular weight bands upon crosslinking of purified B. mori apoLp-III NLP species.
(0.39 MB TIF)

Figure S4 NDGGE of NLP species monitored over time. NLPs incubated at 4°C were analyzed to demonstrate remodeling of individual NLP species. A) Day 1, B) Day 29, C) Day 49, D) Day 70, E) Day 97, F) Day 162.
(0.45 MB TIF)

Figure S5 Direct comparison of NLP remodeling assessment by single molecule analysis (AFM) and bulk analysis (SEC). A–D) Single particle AFM analysis of the distribution of the four purified NLP species. A) NLP-1, B) NLP-2, C) NLP-3, and D) NLP-4. E–H). Bulk SEC analysis derived by integration of the four individual NLP SEC peaks. E) NLP-1, F) NLP-2, G) NLP-3, and H) NLP-4. Only the distribution of NLP species are included in the SEC analysis, not lipid- or protein rich species.
(0.43 MB TIF)

Figure S6 AFM identifies four unique NLP sizes in purified NLP-1 population. Single particle AFM analysis of NLP-1 identifies four unique NLP sizes not elucidated by SEC.
(0.11 MB TIF)
