# Supplementary figures and images for: Isolation, Characterization, and Stability of Discretely-Sized Nanolipoprotein Particles Assembled with Apolipophorin-III
Source: PLoS One. 2010 Jul 19;5(7):e11643. doi: 10.1371/journal.pone.0011643 (PMC2906516; doi:10.1371/journal.pone.0011643)

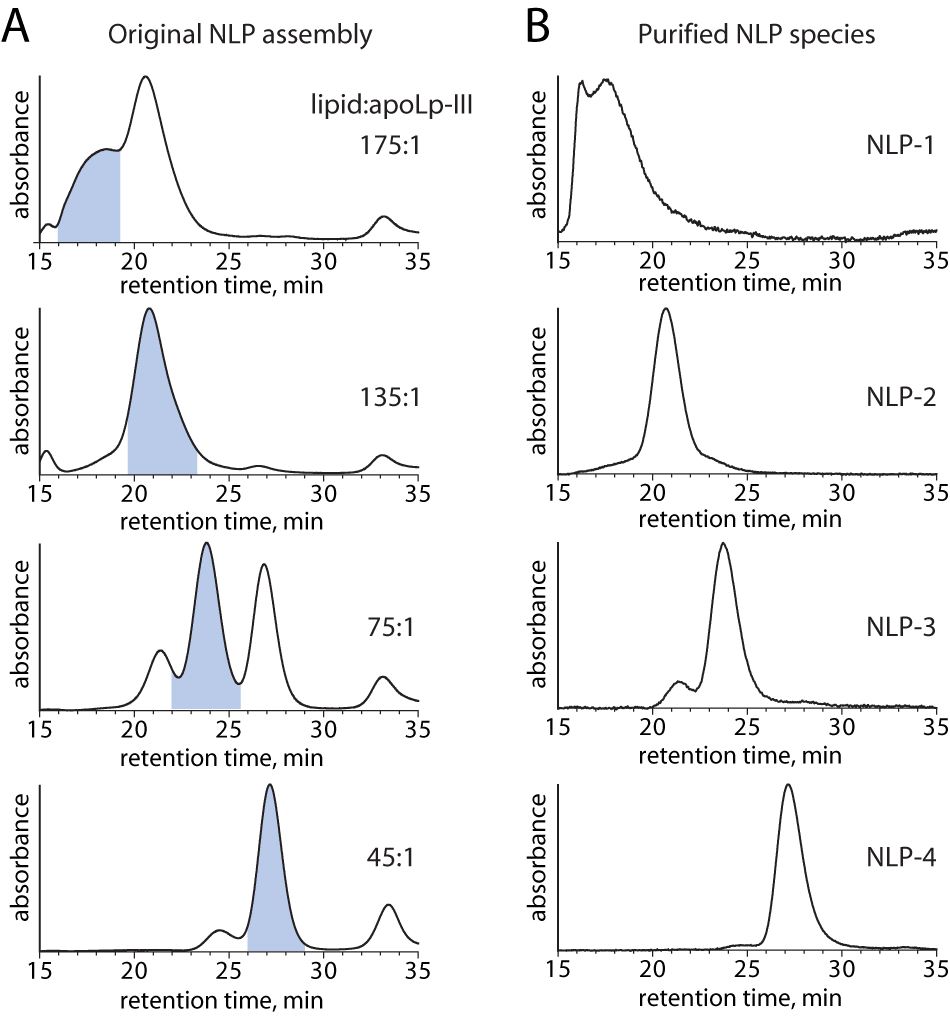

Supplement: Figure S1 — Isolation of B. mori apoLp-III NLP species. A) To isolate the various NLP peaks observed by SEC, four lipid:protein ratios were chosen to provide significant enrichment of the individual peaks (NLP-1, 175:1; NLP-2, 135:1; NLP-3, 75:1; NLP-4, 45:1). SEC fractions (shaded regions) for each NLP peak were pooled for further analysis. B) Pooled fractions from (A) were reanalyzed by SEC, demonstrating purity of the NLP species. (0.20 MB TIF) [file pone.0011643.s001.tif]

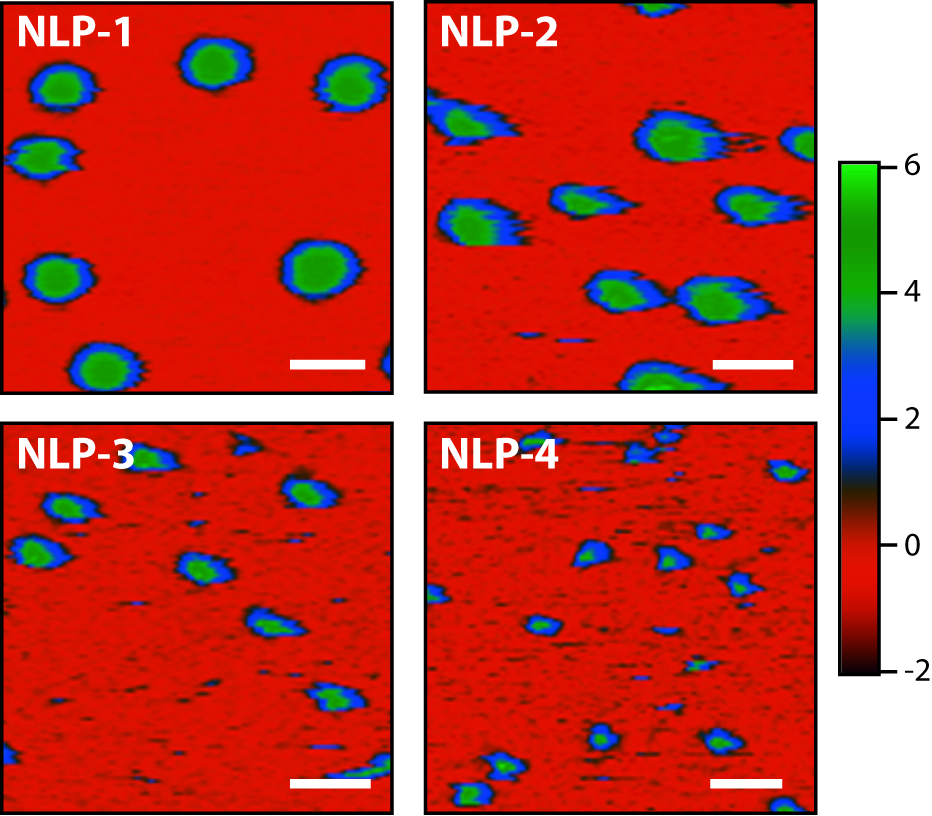

Supplement: Figure S2 — Representative AFM micrographs of the four B. mori apoLp-III NLP species purified by SEC. Scale bars correspond to 50 nm. Full-width half-maximum analysis of NLP diameter is represented by the green area in the pseudo-colored image, which accounts for the tip convolution effect. The slow scan direction (vertical) was used for particle diameter analysis. (1.11 MB TIF) [file pone.0011643.s002.tif]

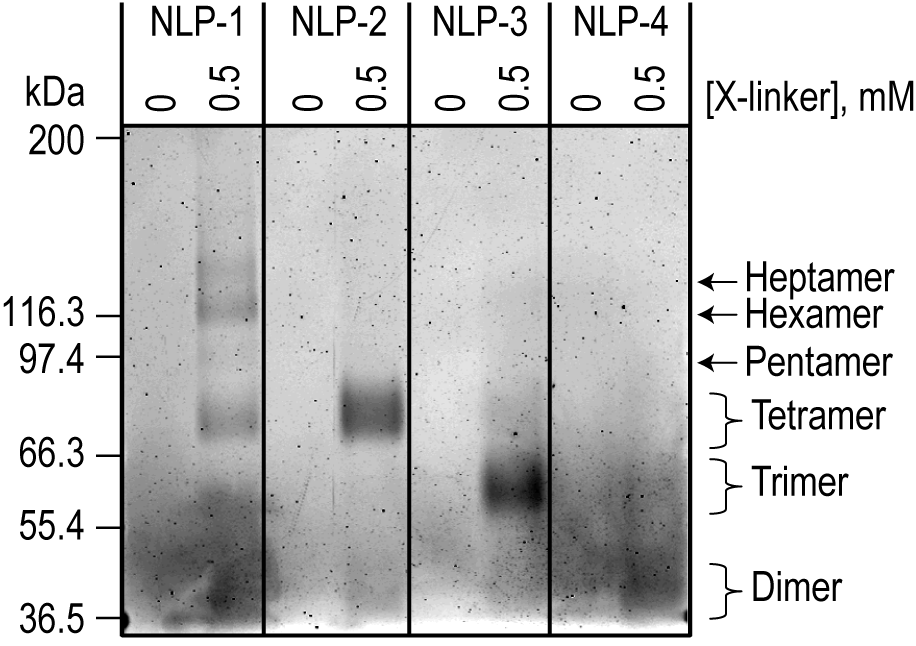

Supplement: Figure S3 — Gel analysis of crosslinking experiments. SDS-PAGE (3–8% Tris-acetate, MES running buffer) was used to resolve high molecular weight bands upon crosslinking of purified B. mori apoLp-III NLP species. (0.39 MB TIF) [file pone.0011643.s003.tif]

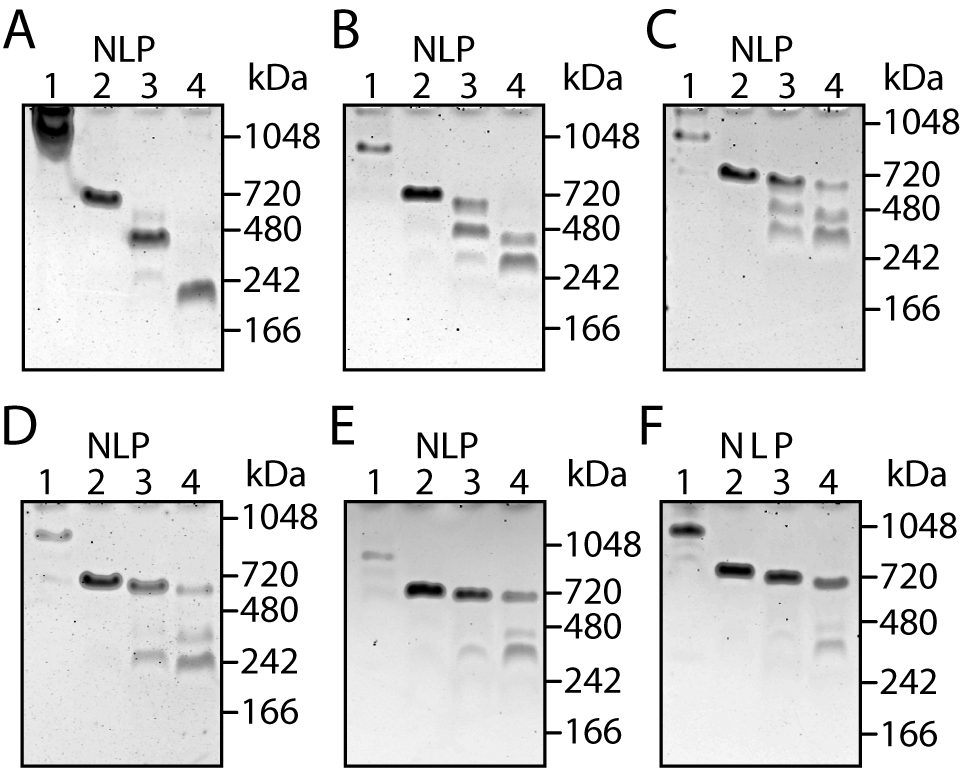

Supplement: Figure S4 — NDGGE of NLP species monitored over time. NLPs incubated at 4°C were analyzed to demonstrate remodeling of individual NLP species. A) Day 1, B) Day 29, C) Day 49, D) Day 70, E) Day 97, F) Day 162. (0.45 MB TIF) [file pone.0011643.s004.tif]

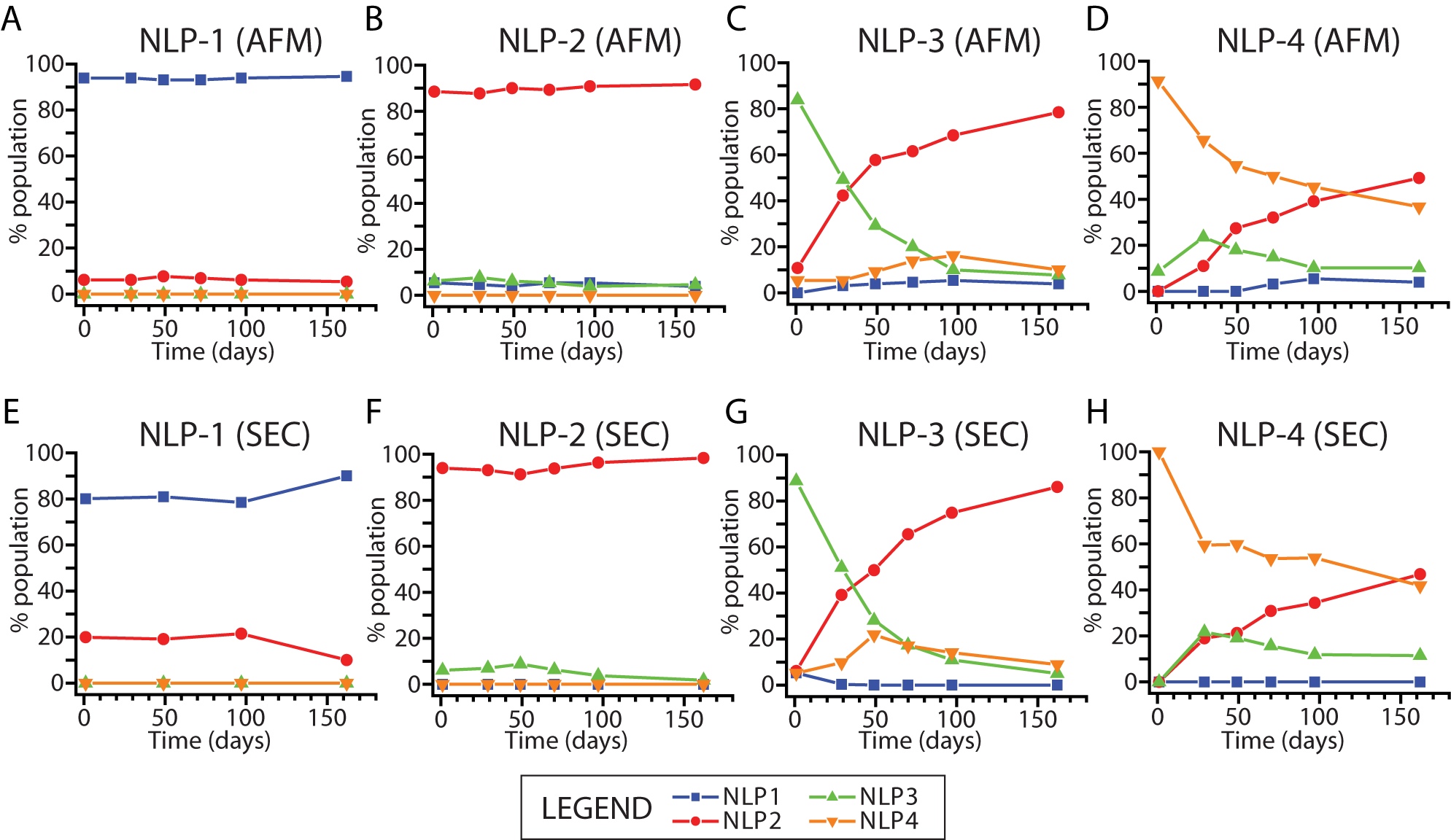

Supplement: Figure S5 — Direct comparison of NLP remodeling assessment by single molecule analysis (AFM) and bulk analysis (SEC). A–D) Single particle AFM analysis of the distribution of the four purified NLP species. A) NLP-1, B) NLP-2, C) NLP-3, and D) NLP-4. E–H). Bulk SEC analysis derived by integration of the four individual NLP SEC peaks. E) NLP-1, F) NLP-2, G) NLP-3, and H) NLP-4. Only the distribution of NLP species are included in the SEC analysis, not lipid- or protein rich species. (0.43 MB TIF) [file pone.0011643.s005.tif]

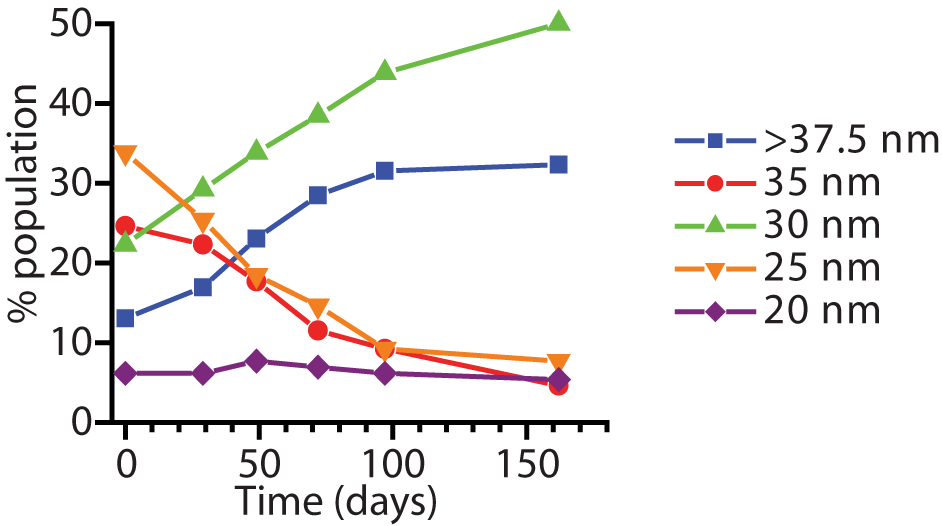

Supplement: Figure S6 — AFM identifies four unique NLP sizes in purified NLP-1 population. Single particle AFM analysis of NLP-1 identifies four unique NLP sizes not elucidated by SEC. (0.11 MB TIF) [file pone.0011643.s006.tif]
